# Whole-genome sequencing combined RNA-sequencing analysis of patients with mutations in SET binding protein 1

**DOI:** 10.3389/fnins.2022.980000

**Published:** 2022-09-07

**Authors:** Li Liu, Xiaoshu Feng, Sihan Liu, Yanqiu Zhou, Xiaojing Dong, Hong Yao, Bo Tan

**Affiliations:** ^1^Department of Gynecology and Obstetrics, The Second Affiliated Hospital of Chongqing Medical University, Chongqing, China; ^2^Institute of Rare Diseases, West China Hospital of Sichuan University, Chengdu, China

**Keywords:** RNA-seq, *de novo*, missense variant, *SETBP1*, clinical diagnosis

## Abstract

SET binding protein 1 (SETBP1) is essential for human development, and pathogenic germline variants in *SETBP1* lead to a recognizable developmental syndrome and variable clinical features. In this study, we assessed a patient with facial dysmorphism, intellectual disability and delayed motor development. Whole genome sequencing identified a novel *de novo* variation of the *SETBP1* (c.2631C > A; p. S877R) gene, which is located in the SKI domain, as a likely pathogenic variant for the proband’s phenotype. RNA sequencing was performed to investigate the potential molecular mechanism of the novel variation in *SETBP1*. In total, 77 and 38 genes were identified with aberrant expression and splicing, respectively. Moreover, the biological functions of these genes were involved in DNA/protein binding, expression regulation, and the cell cycle, which may advance our understanding of the pathogenesis of *SETBP1 in vivo*.

## Introduction

The SET binding protein 1 (*SETBP1*) gene is an oncogene located on the long (q) arm of chromosome 18 at position 12.3. The protein encoded by the *SETBP1* gene contains several motifs and has been shown to bind the SET nuclear oncogene, which is associated with DNA replication and gene expression regulation (Piazza et al., 2018). Mutations in *SETBP1* are involved in multiple diseases, leading to extremely complex genotype-phenotype correlations for the *SETBP1* gene (Acuna-Hidalgo et al., 2017). Somatic mutations of *SETBP1* appear to be gain-of-function mutations and are associated with several hematological malignancies, such as myeloid leukemia (Makishima et al., 2013; Meggendorfer et al., 2013; Piazza et al., 2013; Sakaguchi et al., 2013; Thol et al., 2013; Fabiani et al., 2014; Patnaik et al., 2014; Inoue et al., 2015). In addition, germline loss-of-function mutations in the *SETBP1* gene are correlated with developmental delay, which has a spectrum of symptoms, including absent speech/expressive language delays and mild-severe intellectual disability (Filges et al., 2011). In contrast, germline gain-of-function mutations in the *SETBP1* gene are linked with Schinzel–Giedion syndrome (SGS; OMIM 269150) (Acuna-Hidalgo et al., 2017; Leonardi et al., 2020).

SGS is a rare genetic disorder characterized by characteristic facial features, multiple malformations, and neurological problems (Schinzel and Giedion, 1978; Minn et al., 2002; Al-Mudaffer et al., 2008). Germline *de novo* mutations in the *SETBP1* gene cluster to a hotspot of 12 base pairs in exon 4 of the SETBP1 protein cause SGS (Hoischen et al., 2010). This mutational hotspot is highly conserved and is part of a degron motif targeted by the SCF-βTrCP1 E3 ligase (Piazza et al., 2013). Previous studies have demonstrated that somatic mutations occurring in the hotspot region in the *SETBP1* gene may cause a functional loss of the degron motif, resulting in accumulation of SETBP1 protein in cells and inhibition of the PP2A phosphatase through the SETBP1–SET–PP2A axis (Cristobal et al., 2010; Oakley et al., 2012; Makishima et al., 2013). However, the understanding of the pathogenic mechanism of germline mutations in the *SETBP1* gene is inadequate. Additional clinical and functional investigation is warranted to promote our understanding of the molecular mechanisms of SGS.

Recently, several studies have implemented total RNA sequencing integrated with whole-genome sequencing (WGS) to facilitate interpretation of the pathogenicity of variants by revealing expression and splicing outliers (Kremer et al., 2017; Hollein et al., 2020; Peymani et al., 2022; Yepez et al., 2022). This approach provides an opportunity to explore the molecular mechanisms of germline mutations in the *SETBP1* gene. In this study, we present the clinical characterization of a patient diagnosis as SGS and conducted WGS of parent-offspring trio. The results revealed a novel *de novo* mutation in *SETBP1* that was predicted to be deleterious based on the concordance of generic damage prediction tools. Furthermore, RNA sequencing was performed in this family, and numerous aberrant expression/splicing genes provided supporting evidence for the role of *SETBP1* and insight into the molecular mechanisms of germline mutations in the *SETBP1* gene.

## Materials and methods

### Ethical compliance

Informed consent was obtained from the patient’s parents. This study was approved by the ethics committee of the Second Affiliated Hospital of Chongqing Medical University.

### DNA isolation and whole genome sequencing

We sequenced the patient and her parents following the MGI-2000 protocol outsourced to BGI. Genomic DNA was isolated from peripheral blood using a blood genomic DNA extraction kit (Tiangen Biotech, Beijing, China) in accordance with the manufacturer’s protocol. One microgram of genomic DNA was randomly fragmented by Covaris, and the fragmented DNA was selected by an Agencourt AMPure XP-Medium kit to an average size of 200–400 bp, followed by adapter ligation and PCR amplification. The products were recovered by the AxyPrep Mag PCR clean up Kit. The double-stranded PCR products were heat-denatured and circularized by the splint oligo sequence. The single-strand circle DNA (ssCir DNA) was formatted as the final library and qualified by QC. WGS was performed on the MGI-2000 platform with an average depth of 30x, meaning that the entire genome was sequenced an average of 30 times.

### RNA isolation and sequencing

Total RNA was extracted from peripheral blood and enriched by oligo-dT bead capture, and cDNA was synthesized according to the manufacturer’s protocol. cDNA libraries were constructed using the Illumina trueSeq stranded mRNA sample prep kit protocol (Illumina). Pooled samples were sequenced using a NovaSeq 6000 sequencing system.

### Single-nucleotide variant/INDEL identification, annotation and interpretation

The raw data produced on the MGI-2000 platform were filtered and aligned against the human reference genome (hg19) using the Burrows–Wheeler Alignment tool (Li and Durbin, 2009) after evaluation according to Illumina Sequence Control Software (SCS). The single-nucleotide polymorphisms (SNPs) were called by using Genome Analysis ToolKit software (Van der Auwera et al., 2013).

Variants were annotated using ANNOVAR (Wang et al., 2010). The effects of single-nucleotide variants (SNVs) were predicted by the SIFT, Polyphen-2, and MutationTaster programs. Variants were filtered by a minor allele frequency (MAF) of < 0.1% in the gnomAD (Karczewski et al., 2020), 1000 Genome (Genomes Project et al., 2015), ExAC (Lek et al., 2016) databases and the Exome Variant Server (EVS; NHLBI Exome Sequencing Project).

All variants were interpreted according to ACMG/AMP standards and categorized as pathogenic, likely pathogenic, variants of unknown clinical significance (VUS), likely benign and benign (Richards et al., 2015). Variant validation was performed using Sanger sequencing (ABI 3730xl Genetic Analyzer).

### Copy number variation identification and annotation

Copy number variations (CNVs) were detected by CNVnator, 100-bp bins and standard parameters were used to calculate the read-depth (RD) signal (Abyzov et al., 2011). The CNVs identified were compared with CNVs from the Database of Genomic Variants^1^ to exclude previously reported polymorphisms. The non-polymorphic CNVs were compared with the entries in the DECIPHER,^2^ ISCA,^3^ ClinGen^4^, or ClinVar^5^ databases, evaluated against the literature for known syndromes and overlapping causal aberrations and further analyzed according to the type and size of aberration, function, and expression profile of genes.

#### Quality control for RNA-seq data

Fastp was used to filter low-quality reads from raw sequencing reads to obtain clean reads (Chen et al., 2018b). Then, FastQC and multiQC were used to evaluate the quality of sequencing data, and the average quality score for overall RNA sequences was > 30, indicating that a large percentage of the sequences were high quality (Ewels et al., 2016). DROP v1.2.1 was used to compute the evaluation metrics of mapping with sequencing depth, percentage of mapped reads, and the number of expressed genes (Yepez et al., 2021). The match between the RNA-seq sample and its annotated DNA sample was also determined by DROP with a cutoff of 0.8.

#### Detection of aberrant expression

Aberrant expression was fully detected based on DROP v1.2.1 (Yepez et al., 2021). The clean RNA-sequencing reads were mapped to the human reference genome (hg19) using STAR (2.7.8a) with the Gencode v29 annotation (Dobin et al., 2013). The summarize Overlaps function from the Genomic Alignments R package was used to count reads. To increase statistical power, we performed aberrant expression and splicing analysis by combining our data with 367 blood samples from GTEx data.^6^ Genes with a 95th percentile FPKM (Fragments Per Kilobase of transcript per Million mapped reads) < 1 were considered as lowly expressed in samples and were removed in downstream analysis. In total, nearly 10,000 genes were included. OUTRIDER was applied to identify expression outliers (Brechtmann et al., 2018). Technical and biological covariates, such as sex, age and sequencing batch, were automatically controlled by OUTRIDER, which used an autoencoder implementation. Genes were defined as having aberrant expression with a *p* < 0.01. Reverse transcription-quantitative PCR (RT-qPCR) was performed to validate candidate gene expression.

#### Detection of aberrant splicing

FRASER, which has been included in DROP, was used to obtain splicing outliers (Mertes et al., 2021). Exon–exon and exon–intron junctions with less than 20 reads in all samples were filtered out. In addition, junctions in which the total number of reads at the donor/acceptor splice site was 0 in more than 90% of the samples were also filtered out. Similar to OUTRIDER, FRASER also applies an autoencoder implementation to automatically control the technical and biological covariates. Splicing outlier genes were defined as genes with an adjusted *p* < 0.05. Outlier junctions were defined as those in splicing outlier genes, with an adjusted *p* < 0.05.

#### Pathway enrichment analysis

Functional enrichment of the aberrantly expressed and spliced genes was performed with KOBAS-i, a service that provides comprehensive pathway enrichment analysis using several databases, including GO, KEGG, Reactome, and GWAS catalogs (Bu et al., 2021). An adjusted *p* < 0.1 was selected as the threshold for significant pathways.

## Results

### Clinical features of the patient

**FIGURE 1:**
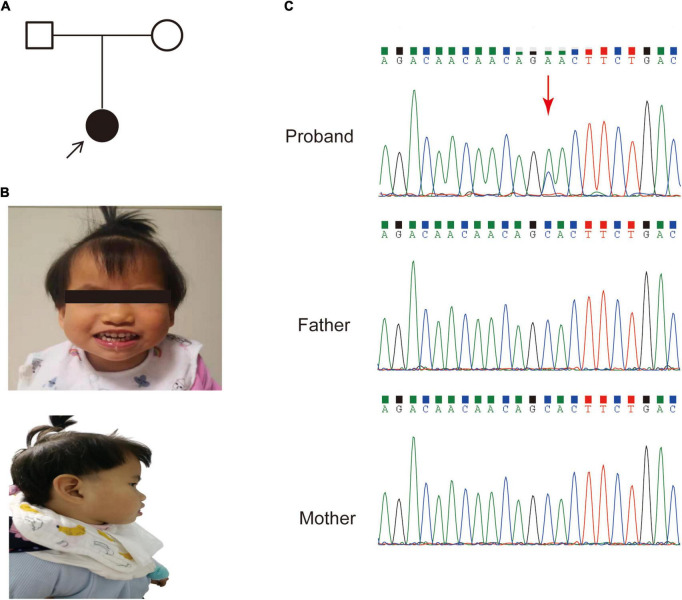
The pedigree and clinical features of the patient. **(A)** Family pedigree of the patient in this study. **(B)** Dysmorphic features, including a prominent forehead, midface hypoplasia and protruding tongue. **(C)** Sanger sequencing of the *SETBP1* gene (c.2631C > A; p. S877R) variant in genomic DNA from the family, confirming that the variant of the SETBP1 gene identified in the patient is *de novo*.

A 3-year-old female was referred to our hospital with global developmental delay, hypertonia and facial dysmorphism. The patient was born after 39 weeks with a normal gestation history. Her parents had no medical history (Figure 1A). She was found to have a motor and language development delay at 2 years old. She had characteristic facial features, including microcephaly, a prominent forehead, midface hypoplasia, a high palatal arch and a protruding tongue (Figure 1B).

Brain MRI at 8 months of age showed delayed myelination of brain white matter and enlargement of the lateral ventricle, and the bilateral frontotemporal extracerebral space was significantly widened. Her karyotype analysis revealed normal results.

### Whole genome sequencing analysis

**FIGURE 2:**
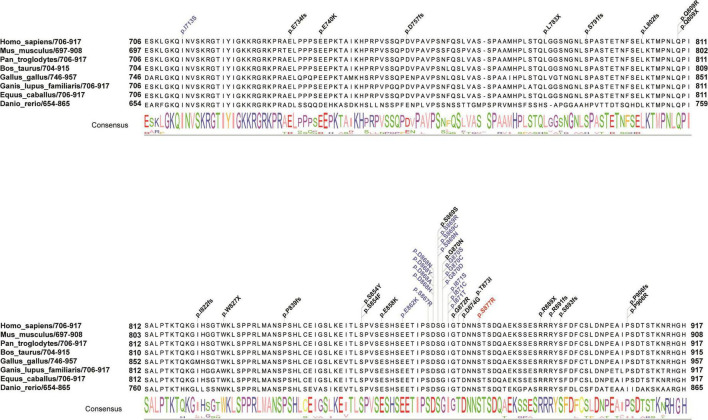
Schematic presentation of the linear SETBP1 protein of the SKI domain with all pathogenic variants. Red indicates the reported variant in the present study that caused an amino acid change (p. S877R) in a highly conserved region. Blue and black indicate variants found in SGS patients and other intellectual disability patients from public data and the literature. The consensus sequence shows the conserved score for each amino acid across eight species. Different colors of the consensus sequence represent different amino acids, and the larger the character, the more conserved the amino acid is.

An average of 102G sequencing data were acquired after WGS for the family member, and no pathogenic CNVs were detected in the proband’s WGS data (Supplementary Figure 1). After variant pathogenicity classification according to ACMG guidelines, one *de novo* missense variant located in the *SETBP1* gene (PS2 + PM2_Supporting + PP3), NM_015559.2: g.42531936C > A, c.2631C > A (NM_000052.7), affected highly conserved residues in close proximity to the canonical region in the SKI domain (p. S877R shown in red in Figure 2), indicating a likely pathogenic variant for the proband’s phenotype. Sanger sequencing confirmed that the c.2631C > A variant was a novel *de novo* variant (Figure 1C and Supplementary Figure 2). In addition, this variant has never been reported in the ClinVar database, HGMD database, or gnomAD database before.

### Transcriptome analysis

**FIGURE 3:**
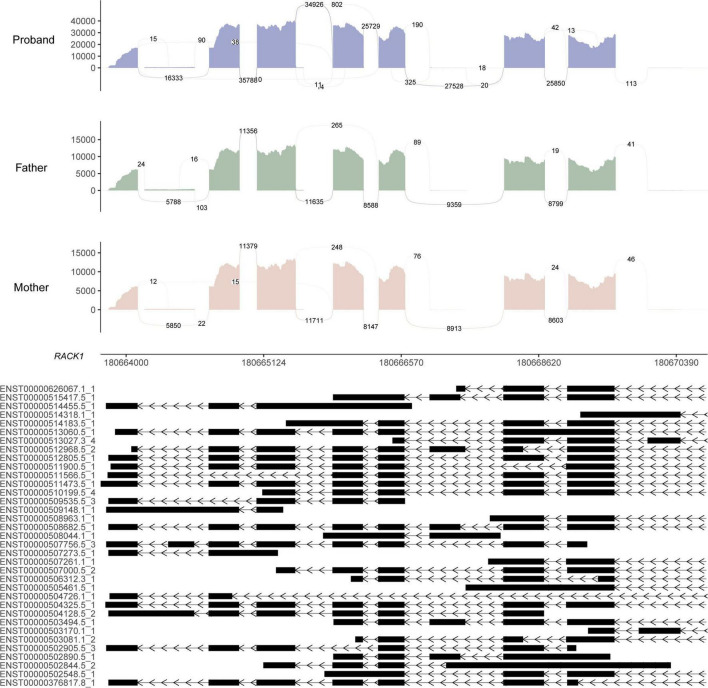
Sashimi plot of the *RACK1* gene. The coverage for each alignment track is plotted as a bar graph. Arcs representing splice junctions connect exons. Arcs display the number of reads split across the junction (junction depth). Genomic coordinates and the gene annotation track are shown below the junction tracks. The aberrant splicing event for the *RACK1* gene in the patient is an alternative acceptor site, which is supported by 325 reads. The X-axis is the genomic region for the *RACK1* gene. The bottom of the figure shows the different transcripts of the *RACK1* gene.

**FIGURE 4:**
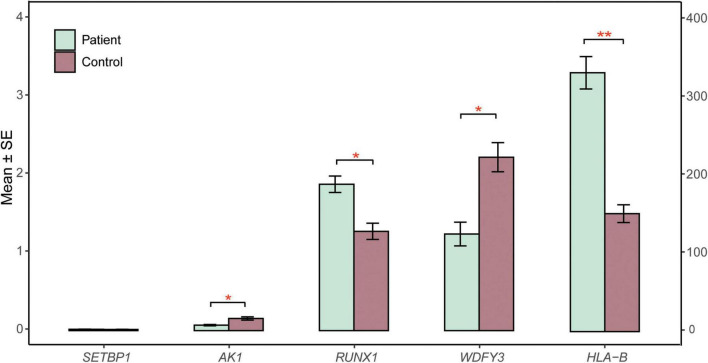
RT-qPCR validation. The relative expression of candidate genes (*SETBP1, WDFY3, AK1, RUNX1, HLA-B*) in patient and control blood cells was quantified by RT-qPCR. β-Actin mRNA was used as an internal control. The experiments were performed in triplicate. Data are shown as the mean ± SE. Two sample *t*-test was performed to test whether the expression of these genes between case and control are significant difference or not. Statistical significance is presented relative to control as *P < 0.05 and **P < 0.01. The *X*-axis on the left presents the mean expression of the *SETBP1*, *WDFY3*, *AK1*, and *RUNX1* genes. The *X*-axis on the right presents the mean expression of the HLA-B gene. SE, standard error.

**FIGURE 5:**
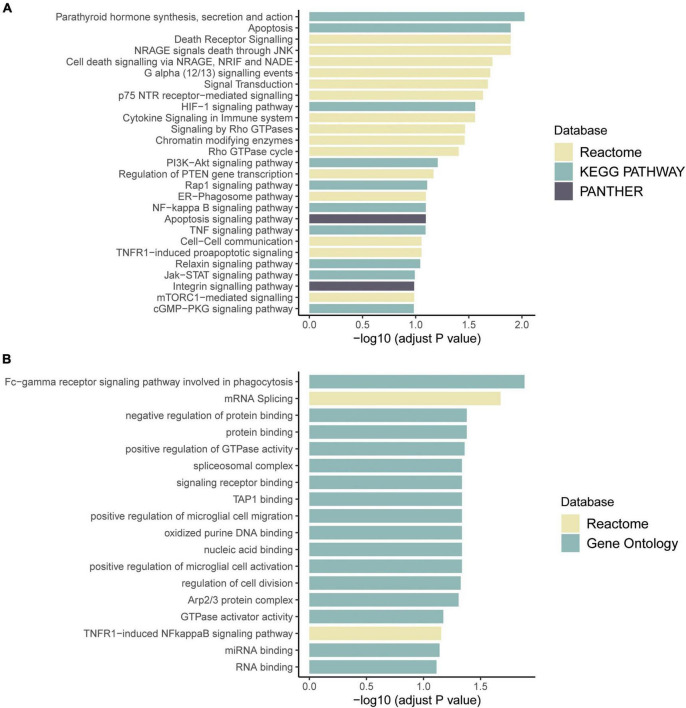
Pathway enrichment results of genes with aberrant expression **(A)** and with aberrant splicing **(B)**. Colors represent pathway items from different databases. The X-axis shows the log-transformed adjusted *p*-value. Pathways with an adjusted *p* < 0.1 were selected as significant pathways and plotted.

RNA sequencing was performed to investigate the potential molecular mechanism of the novel mutation in *SETBP1* (c.2631C > A; p.S877R). Aberrant analysis results and RT-qPCR showed that the RNA expression of the *SETBP1* gene in the patient and control was similar. In total, 77 and 38 genes were identified with aberrant expression and splicing in the patient, respectively (Supplementary Tables 1, 2 and Supplementary Figures 3–8). Several genes directly targeted by SETBP1 or associated with neurodevelopmental disorders (NDDs) have been identified as aberrant genes in patient. For example, the receptor for activated C kinase 1 (*RACK1*) gene and RUNX Family Transcription Factor 1 (*RUNX1*) gene. *RACK1* was a part of the IRE1-RACK1-PP2A complex and was aberrantly spliced in the patient (Figure 3). The *RACK1* gene can modulate neurodegeneration by promoting ERK degradation in Machado-Joseph disease (MJD) and Huntington’s disease (HD) models and participates in the process of neuronal differentiation by regulating *SCN1A* expression (Adams et al., 2011; Dong et al., 2014; Xie et al., 2021). RUNX1 is a direct transcriptional target of SETBP1 and encodes a transcription factor involved in the generation of hematopoietic stem cells and their differentiation into myeloid and lymphoid lines (Vishwakarma et al., 2016). Relative quantification of a subset of genes (*SETBP1*, *WDFY3*, *AK1*, *RUNX1*, and *HLA-B*) by means of RT-qPCR confirmed the accuracy of aberrant analysis with RNA-seq data (Figure 4; two sample *t*-tests; P*_SETBP1_* = 0.485; P*_WDFY3_* = 0.015; P*_AK1_* = 0.037; P*_RUNX1_* = 0.015 and P*_HLA–B_* = 0.004). We next performed enrichment analyses of the aberrantly expressed and spliced genes to delineate the most relevant biological pathways. Functional annotation demonstrated that the biological functions of these genes were involved in DNA/protein binding, expression regulation, and the cell cycle (Figure 5 and Supplementary Tables 3, 4).

### Mutation pattern and genotype-phenotype correlations in the SKI domain of *SETBP1*

To evaluate the correlation between associated variants in the SKI domain and the phenotype of *SETBP1*, a systematic search of original papers was performed (Hoischen et al., 2010; Filges et al., 2011; De Rubeis et al., 2014; Carvalho et al., 2015; Miyake et al., 2015; Takeuchi et al., 2015; Volk et al., 2015; Li et al., 2016; Acuna-Hidalgo et al., 2017; Stessman et al., 2017; Chen et al., 2018a; Daum et al., 2019; Hildebrand et al., 2020; Kurtz-Nelson et al., 2020; Leonardi et al., 2020). A total of 41 variants with *SETBP1*-related NDDs located in the SKI domain were included, including 28 with missense mutations and 13 with loss-of-function mutations (Supplementary Table 5). Overall, variants clustering to a hotspot of 12 base pairs coding for residues 868 to 871 of the SETBP1 protein are known to be associated with severe forms of SGS, possibly through a dominant negative effect (Hoischen et al., 2010). In our study, a novel variant near this region was associated with a similar form of this disease (Figure 2).

## Discussion

In this study, we reported a patient of SGS with severe intellectual disability, developmental delay, epilepsy, hypertonia and distinctive facial dysmorphism. WGS and Sanger validation revealed that these phenotypes may be caused by a novel *de novo* germline missense mutation of the *SETBP1* gene (NM_015559.2: c.2631C > A), which can cause the amino acid change p. S877R (serine - arginine). Due to the extremely low prevalence and great phenotypic heterogeneity of SGS, it is difficult to recognize by clinicians and is usually diagnosed based on the reminiscent clinical features (Acuna-Hidalgo et al., 2017). In this case, an accurate genetic diagnosis significantly improved the management of the patient and reproduction of this family.

Our results revealed that germline *de novo* heterozygous missense variants adjacent to the mutation hotspot of the *SETBP1* gene tend to cause atypical SGS. Previous studies have shown that germline *de novo* mutations in the *SETBP1* gene cluster to residues 868–871 of the SETBP1 protein, which are associated with severe forms of SGS (Hoischen et al., 2010). In this study, the *de novo* variant identified in this patient is located in residue 877, which is close to the mutation hotspot of the *SETBP1* gene. Some phenotypes in this patient were mild relative to typical SGS patients, including ventriculomegaly, skeletal abnormalities, and hydronephrosis. In addition, several individuals with atypical SGS carrying heterozygous missense variants outside the mutation hotspot have been reported. Acuna-Hidalgo et al. (2017) identified four individuals carrying *SETBP1* variants in close proximity to the canonical mutation hotspot, including p.(Glu862Lys), p.(Ser867Arg), and p.(Thr873Ile), who showed a milder developmental phenotype with clinical characteristics that partially overlapped with classical SGS. Moreover, individuals with variants located further from the mutation hotspot showed a variable clinical phenotype ranging from mild to severe intellectual disability (Leonardi et al., 2020; Wong et al., 2022). These findings highlight that the variable severity of broad clinical features depends on the proximity of variants to the mutation hotspot.

Integrative analyses identified that the missense variant reported in this study likely disrupts SETBP1 protein functions via mechanisms including DNA/protein binding, transcription and the cell cycle. While the transcription of the *SETBP1* gene was not affected, it is possible that this missense *SETBP1* mutation has a subtle but distinct effect on the regulation, since 77 aberrantly expressed and 38 spliced genes have been identified in the patient. Consistent findings have been reported by other researchers. In a recent paper, Wong et al. revealed through cellular experiments that classical SGS variants located in the mutation hotspot showed increased protein stability and higher SETBP1 protein levels, while *SETBP1* variants outside the mutation hotspot disrupt DNA binding and transcription independent of protein abundance (Wong et al., 2022). Future studies that delineate the structural impact of *SETBP1* variants and how they affect interactions with other genes will contribute to the understanding of their impacts on protein functions and thus etiology.

Taken together, our findings expand the current understanding of the genetics and clinical spectrum of *SETBP1* variants. In addition, by integrating WGS and RNA-seq analyses, we provide insight into the pathogenicity of a germline *de novo SETBP1* variant in a patient diagnosed with atypical SGS.

## Data availability statement

The original contributions presented in this study are included in the article/Supplementary material, further inquiries can be directed to the corresponding author/s.

## Ethics statement

The studies involving human participants were reviewed and approved by the Ethics Committee at The Second Affiliated Hospital of Chongqing Medical University. Written informed consent to participate in this study was provided by the participants’ legal guardian/next of kin. Written informed consent was obtained from the individual(s), and minor(s)’ legal guardian/next of kin, for the publication of any potentially identifiable images or data included in this article.

## Author contributions

LL performed the experiments and wrote the manuscript. XF and SL performed RNA-seq analysis and wrote the manuscript. YZ and XD collected the clinical information of the patient and her parents. HY and BT designed and supervised the study and reviewed the manuscript. All authors contributed to the article and approved the submitted version.

## Footnotes

1
http://projects.tcag.ca/variation/

2
http://decipher.sanger.ac.uk/

3
http://www.iccg.org/

4
https://search.clinicalgenome.org/kb/gene-dosage

5
http://www.ncbi.nlm.nih.gov/clinvar/

6
https://zenodo.org/record/5638707#.Yt4B63ZByUk
