# Supplementary material for: Whole-genome sequencing combined RNA-sequencing analysis of patients with mutations in SET binding protein 1
Source: Front Neurosci. 2022 Sep 7;16:980000. doi: 10.3389/fnins.2022.980000 (PMC9490002; doi:10.3389/fnins.2022.980000)
Supplement: Supplementary file 1 [file Data_Sheet_1.docx]

Supplementary Material

# Supplementary Figures and Tables

##
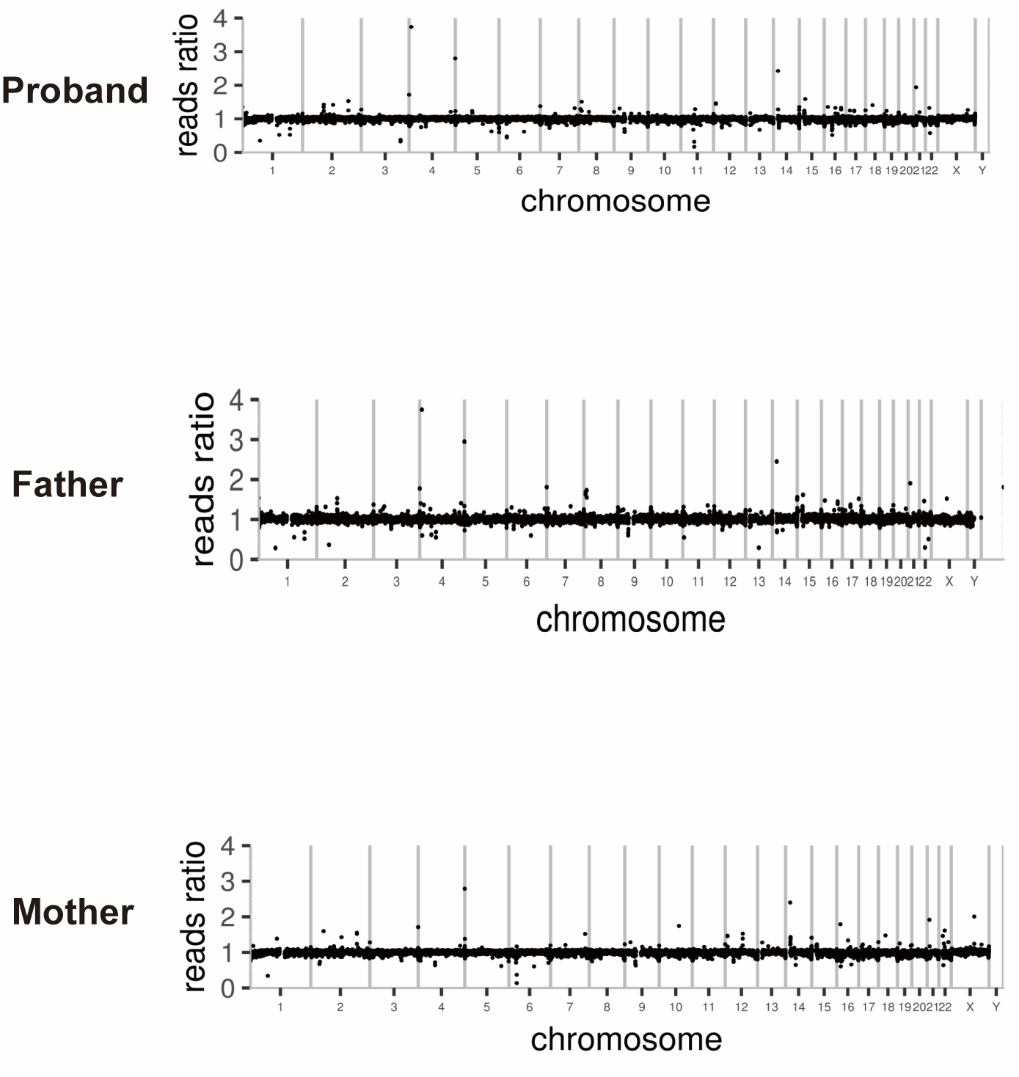
Supplementary Figures

**Supplementary Figure 1.** Copy number variant (CNV) results detected by CNVnator in the patient and her parents. Each dot presents a region with 100-bp bins. Read ratio is detected by comparing the coverage of regions in the sample and control projects.


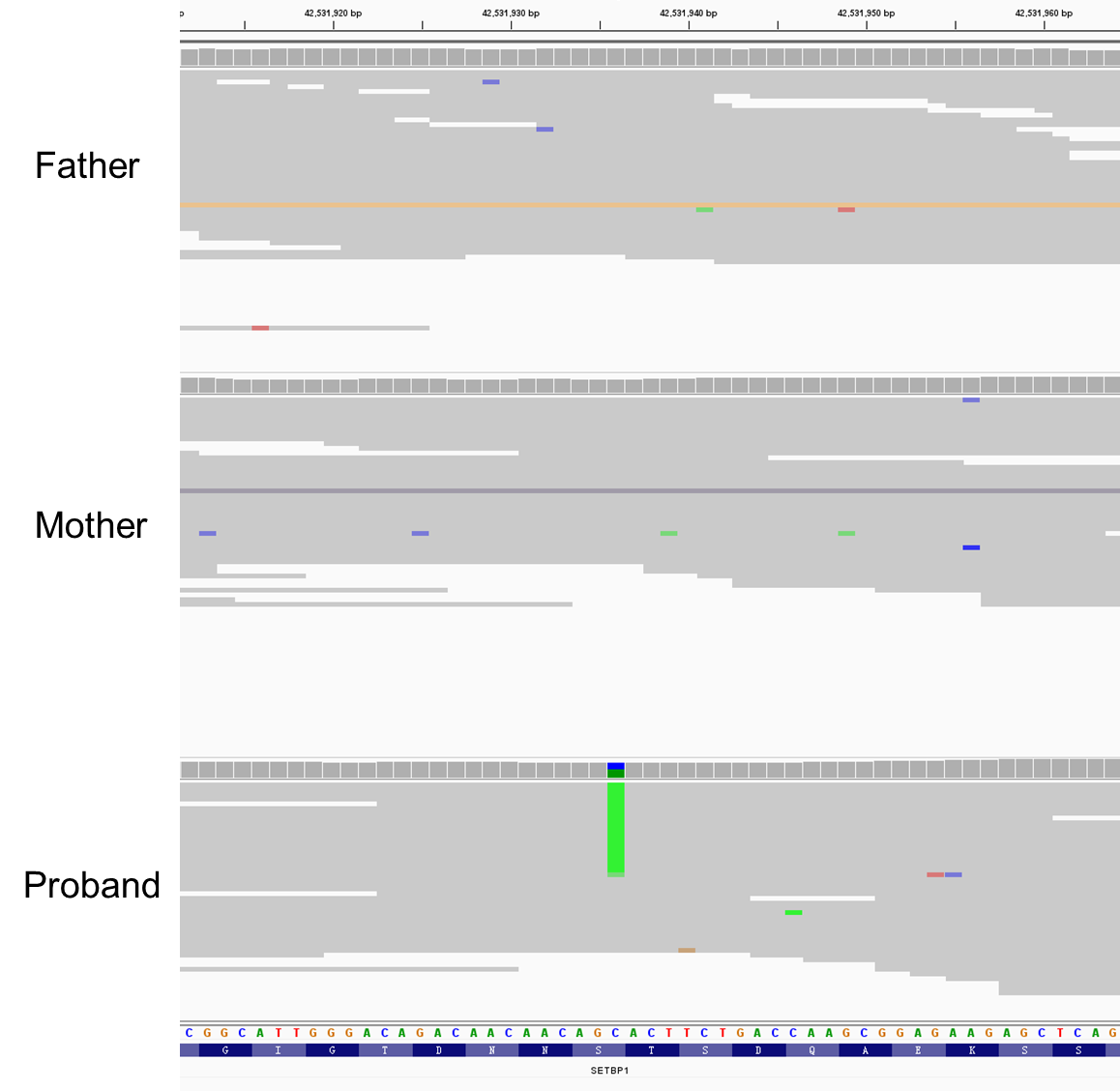


**Supplementary Figure 2.** *SETBP1* whole genome sequence identifies c.2631C>A in IGV. The red arrow pointed to the variant site, supported by numerous aligned reads. The result shows that this variant was de novo and absent in the patient’s parents.


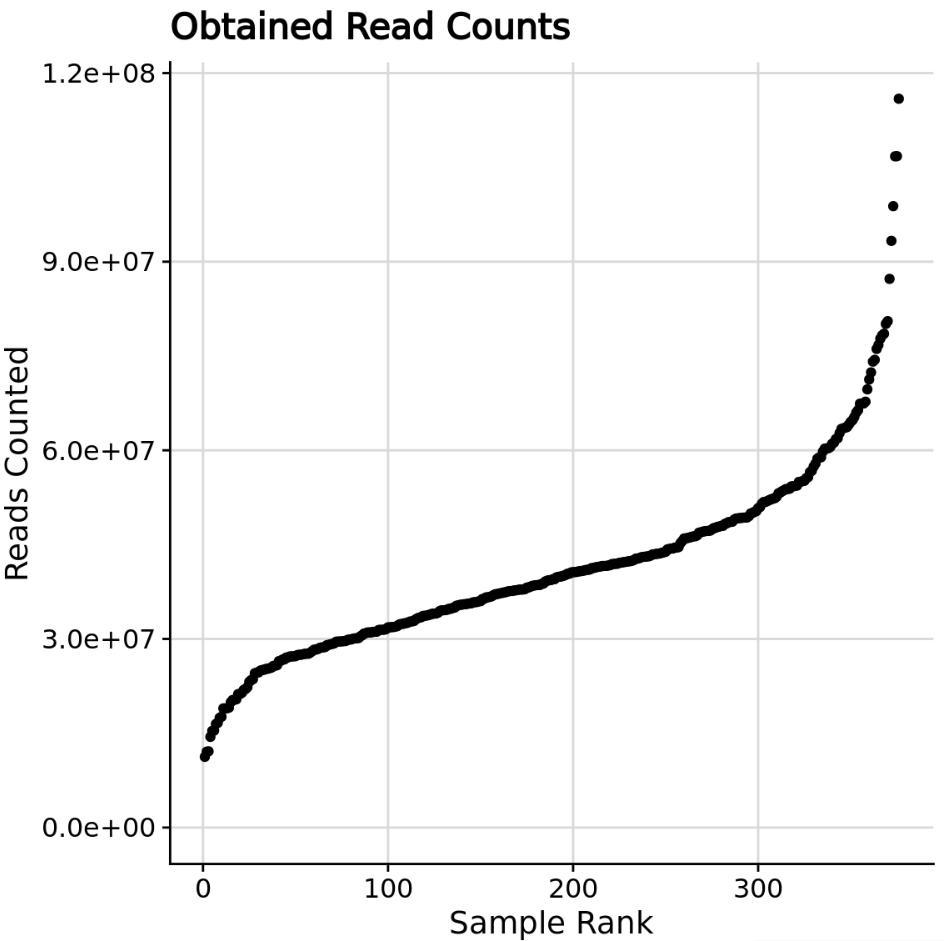


**
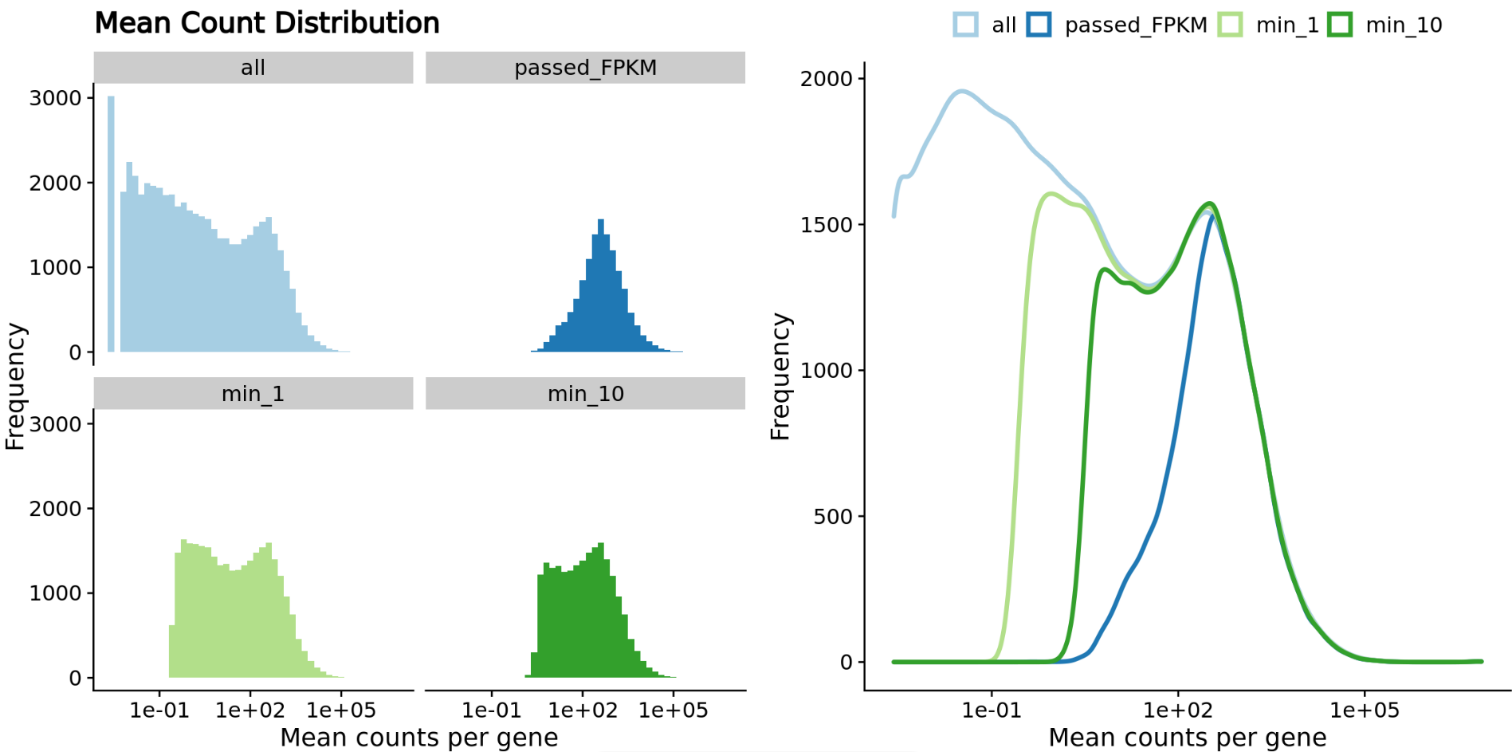
Supplementary Figure 3.** Read counts obtained from each sample. By integrating blood samples from GTEx data, the sample size in our analysis was 376. The read count for each sample ranged from 15M to 130M.

**Supplementary Figure 4.** Mean count distribution for each gene across samples. Passed_FPKM indicates the genes were passed for fragments per kilobase of transcript per million mapped reads filtering. Min_1 indicates genes with the lower expression (1%) among all genes. Min_10 presents genes that have a lower expression (10%) among all genes.


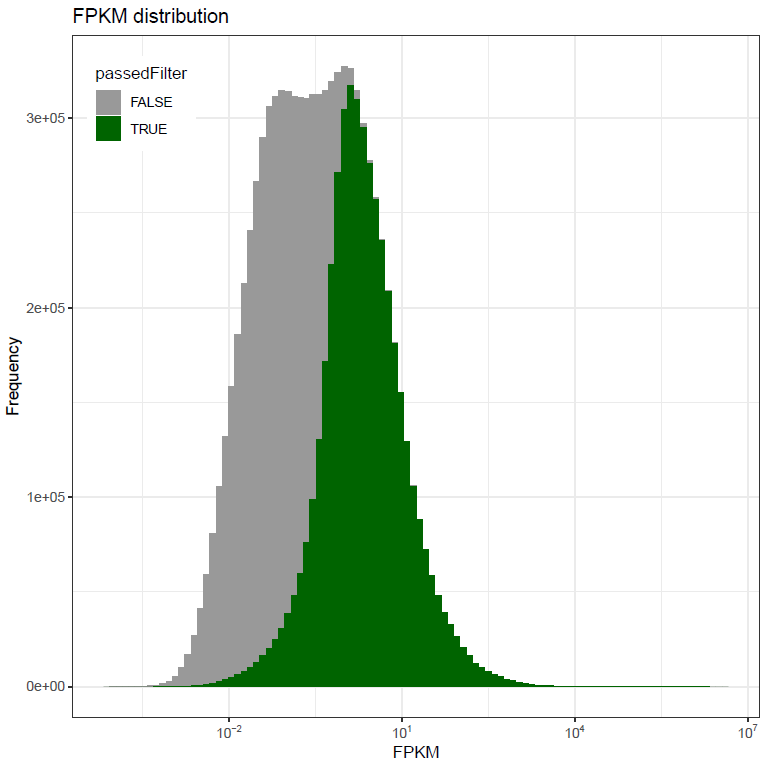

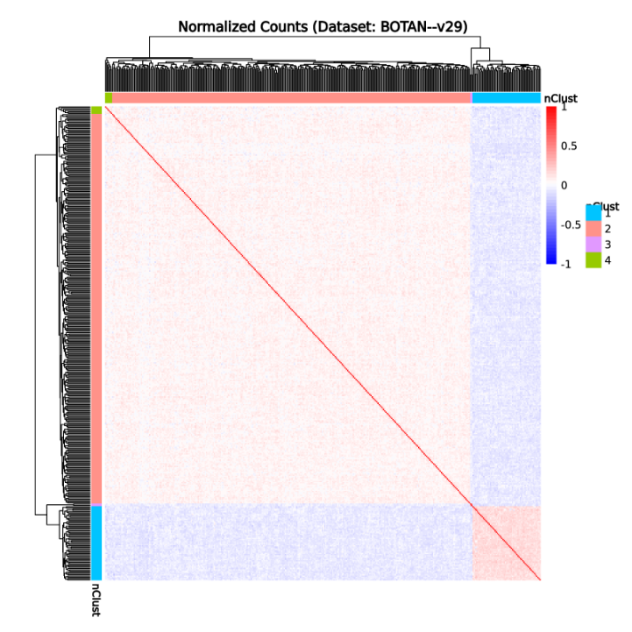

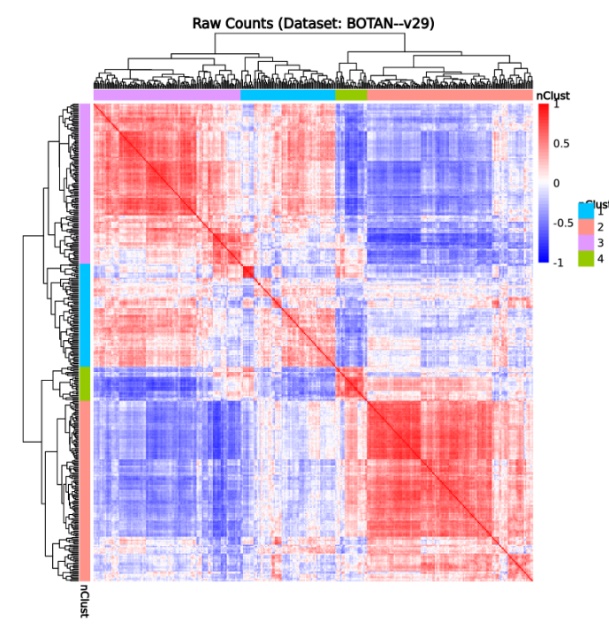
**Supplementary Figure 5.** Retaining genes with FPKM>1 in at least one sample. In total, 48232 genes did not pass the filter. FPKM: fragments per kilobase of transcript per million mapped reads

A

B

**Supplementary Figure 6.** Heatmap plot of gene expression in each sample before (A) and after (B) adjust confounding factors, including technical and biological covariates.


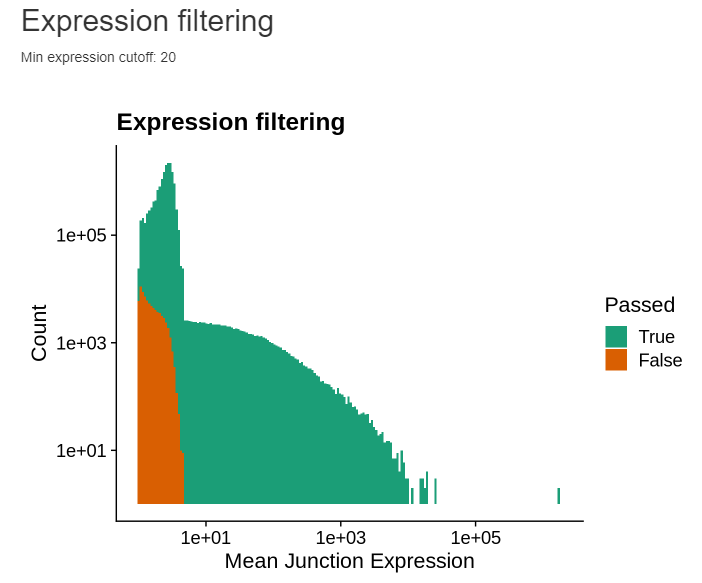


**Supplementary Figure 7.** Distribution of read counts in each junction across samples. Exon–exon and exon–intron junctions with less than 20 reads in all samples were filtered out. In addition, junctions in which the total number of reads at the donor/acceptor splice site was 0 in more than 90% of the samples were also filtered out.


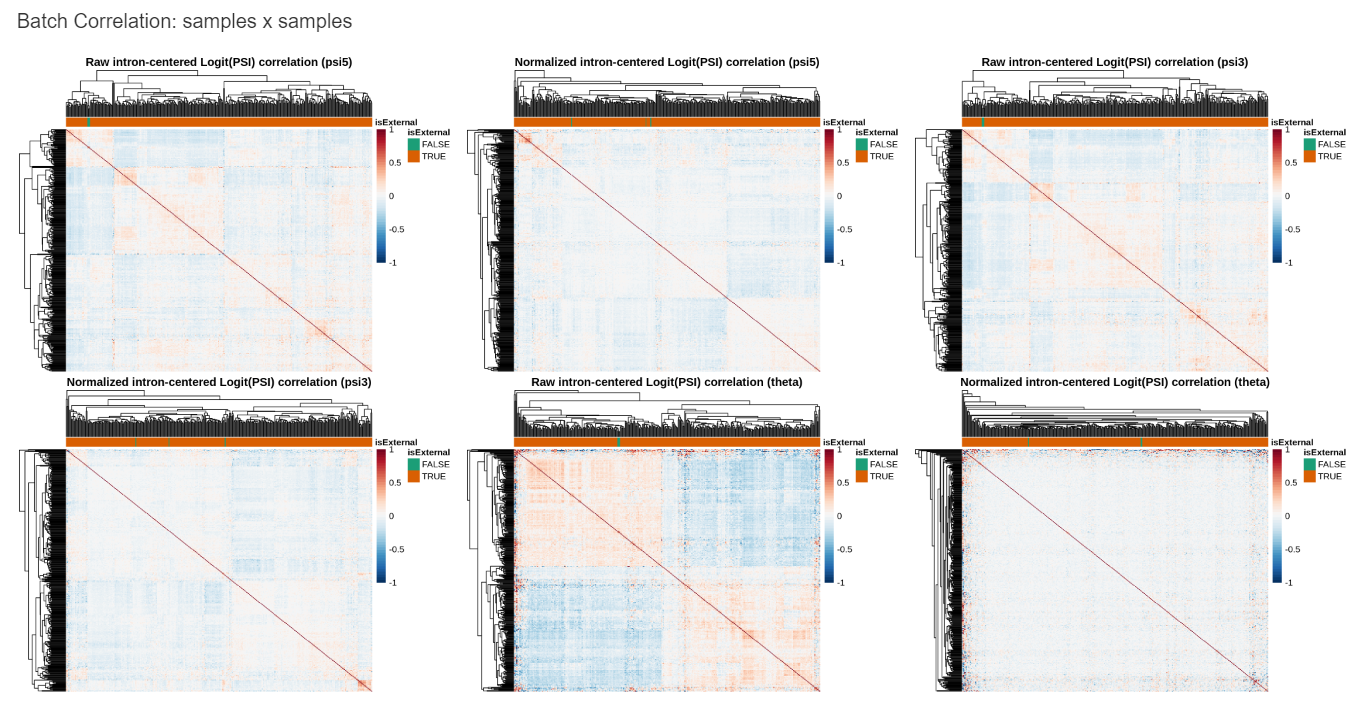


**Supplementary Figure 8.** Batch correlation across samples in aberrant splicing analysis with intron-centered logit (PSI). The type of PSI includes psi5, psi3, and theta, which stand for the splicing metrics of alternative acceptors (psi5), alternative donors (psi3), and splicing efficiencies at donors and acceptors (theta), respectively.
